# Decentralization Matters: Association of Adherence to Treatment and Distance for the Management of Non-Communicable Diseases in Rural Tanzania

**DOI:** 10.3390/ijerph21111506

**Published:** 2024-11-13

**Authors:** Paolo Belardi, Noemi Bazzanini, Francesca Cera, Katunzi Mutalemwa, Francesca Tognon, Emmanuel Ndile, Alessandro Mele, Rehema Itambu, Rhoda Naftali, Bernard Kakala, Veronica Kayombo, Benjamin Mfaume, Bruno Ndunguru, Samwel Marwa, Mario Saugo

**Affiliations:** 1 Doctors with Africa CUAMM, Iringa P.O. Box 11, Tanzania; 2 Doctors with Africa CUAMM, 35121 Padua, Italy; 3 Muhimbili National Hospital, Dar-es-Salaam P.O. Box 65000, Tanzania; 4 Tosamaganga Regional Referral Hospital, Iringa P.O. Box 11, Tanzania; 5 Iringa District Council, Iringa P.O. Box 108, Tanzania

**Keywords:** non-communicable disease, NCD, Tanzania, diabetes, hypertension, decentralization, health service development

## Abstract

Since March 2019, a non-communicable diseases program has been established at hospital level, with enrollment and clinical reassessment every 6 months. Since July 2023, monthly enrollment and visits have also been conducted at health center level. This study aimed at assessing the adherence to scheduled follow-up visits following the decentralization of the integrated NCDs program from Hospital to Health Center level and investigate factors influencing follow-up adherence. The study was performed in a rural district in Iringa Region, Tanzania. Adherence was measured at both levels. Multivariate regression analysis was conducted to describe socio-demographic and clinical factors influencing attendance at the 6-month hospital-level visit. Among 2198 patients enrolled at the hospital level, weighted adherence over 42 months was 40.8% (95% CI 39.0–42.6%) at the 6-month visit. Multivariate analysis revealed that as the distance from the hospital increased, the probability of attendance decreased (OR 0.17; 95% CI: 0.08–0.39). Among 571 patients enrolled at the residence level, adherence over the first 10 months of program implementation was 91.6% (90.4–92.8%). The findings showed that distance was by far the most important barrier to follow-up adherence and suggested that decentralizing the program from the hospital to peripheral health centers may ensure high follow-up rates.

## 1. Introduction

Cardiovascular diseases (CVD) represent one of the major public health challenges worldwide [1,2]. Globally, 17.9 million people were estimated to die from cardiovascular diseases in 2019, accounting for 32% of all deaths [3]. Of these, 85% were attributed to myocardial infarction or strokes [3,4]. Notably, more than three-quarters of CVD deaths occur in low- and middle-income countries (LMIC) [5]. In Sub-Saharan Africa (SSA), the total number of deaths due to CVD across all age groups exceeded one million in 2019, marking a 78.0% increase compared to 1990 [6]. While CVD mortality rates have declined in most regions over the past 40 years—largely due to advancements in early detection and the treatment of hypertension (HTN), diabetes mellitus (DM), and hypercholesterolemia—LMICs continue to bear the brunt of the burden. An estimated 1.28 billion people aged 30–79 worldwide are affected by HTN, with nearly two-thirds residing in LMICs. Alarmingly, 46% of adults with HTN are unaware of their condition [5].

According to the international literature, CVDs-related death rates have been 2.1 times higher in SSA Countries than in High-Income Countries (HIC) [6]. This disparity has been largely driven by increasing urbanization and the adoption of Western lifestyles, which have led to higher body mass index (BMI), blood pressure (BP), and fasting blood glucose (FBG) levels [7]. Between 1990 and 2019, the number of DM cases in SSA increased by 131.7%, with a 2.1% rise in age-standardized prevalence rates [8]. While elevated BP prevalence has steadily declined in HICs since the 1970s, it has continued to rise in SSA, resulting in a doubling of deaths attributable to high BP [9]. The World Health Organization (WHO) reports a higher prevalence of HTN in Africa, with approximately 46% of adults aged 25 years and older, compared to 35% in the Americas and other developed countries, and 40% in other parts of the world [10,11]. Non-communicable diseases (NCDs) are becoming the leading cause of death in Africa, with the percentage of deaths rising from 24% in 2000 to 37% in 2019. By 2021, 24 million people in the region were living with DM, and this number is projected to increase by 129% to 55 million by 2045 [12].

NCDs are a significant public health issue in Tanzania. In 2021, the age-standardized death rate for the four main NCDs, CVD, chronic respiratory disease, cancer, and diabetes was 557 per 100,000 in men and 498 per 100,000 in women [13]. Tanzania has made progresses on NCD regulations such as tobacco taxes, national policies and guidelines. However, progress has been limited in other areas such as alcohol control, salt and trans fat consumption and physical activity guidelines [13,14,15]. HTN is the most common NCD and impacts approximately 25% of the adult population, representing the leading cause of death after HIV [13]. Diagnosed individuals often are neither in BP treatment nor seeking care [15,16,17]. Specifically, rural populations are less likely to be aware of their HTN and are, therefore, less likely to be on treatment [18,19,20,21]. DM also plays a significant role, with a prevalence of 10.3% among adults aged 20–79, accompanied by a high incidence of complications [22].

In SSA, healthcare systems struggle to provide continuous long-term care for patients affected by HTN and DM [15,16,18,23], including limited healthcare infrastructure, healthcare workforce shortages, limited access to medications, lack of awareness, and education, cultural, and social factors [24,25,26,27,28]. Additionally, the 2012 WHO STEPs survey revealed that three-quarters of participants with HTN or DM had never been previously diagnosed, and less than half of those with a known diagnosis were receiving care [18,19,21]. International studies suggested that individuals from lower socioeconomic backgrounds and those living in rural areas are less likely to have their blood glucose or BP measured regularly [16,17,29,30]. The factors associated with low adherence rates to treatment and follow-up care are mainly due to social, cultural and economic conditions, as demonstrated in several studies in LMICs [31,32,33,34,35].

In 2010, the WHO launched the Package of Essential NCD Interventions (WHO PEN) to facilitate the decentralization of healthcare services to primary care settings [36]. Particularly, this approach aimed to provide accessible treatment at community health centers (HC) for common NCDs through an integrated outpatient care model, which has been scaled up to different countries [36,37,38]. In March 2019, an integrated management system of HTN and DM was implemented in rural Tanzania. In the first phase of the program, each patient underwent initial medical assessment at the hospital level [39]. According to the WHO dedicated package, reassessment visits were conducted at the hospital, every six months [40]. In July 2023, based on the PEN PLUS approach, a significant change in the program occurred with the launch of the second phase, shifting enrollment and routine follow-up to peripheral HCs.

Despite numerous efforts and initiatives at macro level, a limited number of studies have evaluated the effects of decentralizing HTN and DM care from hospitals to peripheral health facilities in SSA [41,42,43,44].

To contribute to addressing the gap in evaluating the impact of decentralizing NCDs interventions to lower-level health facilities, this study aimed at assessing the adherence to the scheduled follow-up visits following the decentralization of the integrated NCDs program from the hospital to the HC level. Additionally, this study illustrated the factors influencing adherence to follow-up care for patients enrolled during the first five year of the integrated NCDs Program at Hospital level, which in turn contributed to the decision to decentralize the clinical activities to the lower-level facilities.

## 2. Materials and Methods

### 2.1. Study Design

This is a retrospective observational study on adherence to follow-up among hypertensive and diabetic patients in rural Tanzania.

### 2.2. Study Setting

Iringa District Council (Iringa DC) is located in a rural area 500 km southwest of Dar es Salaam, with a population of around 330,000 inhabitants spread over a surface area of about 20,000 km^2^ [45]. Iringa DC is one of the five districts of the Iringa region, which has a total population of 1,192,728 people [45]. The majority of inhabitants are peasants, others own businesses, and only a handful have formal jobs. The local healthcare system of Iringa DC includes Iringa DC Public Hospital, Tosamaganga Regional Referral Hospital (TRRH), 10 HCs, and 103 dispensaries serving 134 sparsely populated villages. Particularly, TRRH is a not-for-profit faith-based hospital, which counts 192 beds, recently recognized as Referral Hospital at Regional Level. The 10 HCs are spread and serve as peripheral referral centers within the reference territory.

### 2.3. The NCDs Program

Starting in October 2016, Doctors with Africa CUAMM and the Iringa DC established an outpatient service for individuals with NCDs at the TRRH outpatient department. In March 2019, an integrated management system for HTN and DM was commenced following the completion of the Protocol of Cooperation Agreement among the Iringa DC, TRRH, and the international NGO Doctors with Africa CUAMM [39]. The overarching goal was to ensure access to quality and equitable healthcare for the district’s population. The agreement was designed to enhance and strengthen the healthcare system in Iringa DC, specifically focusing on the prevention and treatment of HTN and DM at both the Hospital and HC levels.

The initial protocol included an assessment at TRRH, where patients underwent medical visits, BP and finger-stick FBG measurements, along with laboratory exams (creatinine, in DM patients and total cholesterol in selected cases). After receiving their diagnosis from a medical doctor and health education from a nutritionist, patients were provided with a personalized treatment card (TC). Patients were required to return to the hospital for a reassessment visit every six months (±1 month), following guidelines provided by the WHO for a dedicated NCDs package of care [40]. Further visits could also have been performed at the closest HC for drug refilling, additional BP, and finger-stick FBG controls, if needed. Clinical and treatment information were documented in the TCs at each reassessment visit at TRRH. This organizational setting is referred to hereafter as the TRRH Program.

In July 2023, a major change was implemented: enrollment (medical visit, BP, and finger-stick FBG measurements) and supervision visits were routinely conducted in nine HCs. After enrollment, a thorough clinical assessment at TRRH was recommended for patients with HTN or DM (medical visit, BMI and BP measurements, serum FBG, and HbA1c for those with diabetes), but excluding those above 80 years old and those who declared financial or transportation constraints for traveling to TRRH. Supervision visits at the HCs were scheduled every month, while reassessment visits at the TRRH are considered for selected patients (e.g., resistant HTN, diabetic decompensation, established organ damage in adult and elderly young patients). This setting is referred to hereafter as the HC Program.

Screening, diagnosis, and treatment of HTN and DM as well as criteria for referral to a higher level of care during follow-up were provided according to national NCDs guidelines [46].

### 2.4. Inclusion and Exclusion Criteria

Patients aged 18 years and above, diagnosed with both new and known HTN and/or DM, who were enrolled in the TRRH program between March 2019 and March 2024, and in the HC program between July 2023 and March 2024, were included in the study. Pregnant women were excluded.

The outcome measures included in the study were the following:

- for patients enrolled in the TRRH program: adherence to scheduled reassessment visits at TRRH at 6, 12, 18, 24, 30, 36, and 42 months;
- for patients enrolled in the HC program: adherence to scheduled supervision visits at the HCs at 1, 2, 3, 4, 5, 6, 7, 8, 9, and 10 months.

Adherence was considered as the completion of the visit within the deadline scheduled in the previous visit. In each program, a delay of up to one month was considered acceptable.

### 2.5. Data Collection and Covariates

Data were collected retrospectively from the hospital’s electronic medical records and HCs registers. The healthcare staff on duty were responsible for collecting data on patients’ Enrollment and TCs. The information was verified by a Medical Doctor before being entered anonymously into a specifically designed database using the software EpiInfo v. 7.2.3 [47].

The data collection forms for patients visited within the TRRH program included socio-demographic factors (such as age, gender, occupation, health insurance status, and distance from the TRRH) and clinical factors (previous/new diagnosis of HTN or DM, previous diagnosis of cardiovascular diseases such as myocardial infarction, stroke, heart failure, renal failure according to eGFR results, diabetic foot or amputation, lifestyle factors such as alcohol consumption, smoking, and physical inactivity, previous registrations of BP or FBG if any, BP, height, weight, body mass index (BMI), finger-stick FBG, and HbA1c for patients with Diabetes Mellitus). We considered CVD complications at baseline if there were not (i) previous medical diagnosis of myocardial infarction, stroke or heart failure; (ii) previous medical diagnosis of diabetic foot or amputation in DM patients; (iii) chronic kidney disease (CDK) stage ≥ 3a according to the baseline creatinine and estimation of eGFR (through CKD-EPI equation). The lifestyle was considered according to the clinical history: Alcohol abuse (Y/N), Sedentary (Y/N), Smoking (Y/M).

The data collection forms for patients enrolled in the HC program comprised the following variables: age, gender, health insurance status, distance from the hospital, and essential clinical data recorded at each visit (BP; height, weight, BMI in obese patients; finger-stick FBG in obese or symptomatic patients; pharmacological treatment at each visit). BP was measured using a manual sphygmomanometer, after five minutes of rest, in a seated position, with the arm at heart level. Elevated values were confirmed twice. Patients were classified as hypertensive if a BP of ≥140/90 mmHg was recorded on two separate occasions. FBG levels, either fasting (after at least 8 h since the last meal) or random, were measured using a GlucoPlus™ glucometer, GLUCOPLUS INC. 2323 Halpern, St-Laurent (Montreal) Québec, Canada H4S 1S3.

### 2.6. Statistical Analysis

Socio-demographic and clinical variables were dichotomized or grouped into classes, namely sex, age, distance from the TRRH (0–19/20–39/40–59/60–79/80+ km), health insurance status, diagnosis, disease awareness, (<140/90, ≥140/90, ≥160/100, ≥180/110 mmHg), FBG (<7, ≥7, ≥9, ≥11 mmol/L). Among patients enrolled at the TRRH, occupation (other/peasant/skilled workers), lifestyle (correct/uncorrect), complications (yes/no), and BMI (<18, 18–24, 25–29, ≥30 kg/m^2^) were also recorded.

The proportion of adherence along the subsequent visits was considered separately for those enrolled at the TRRH (6-month reassessment visits) and those enrolled at the HC level (1-month supervision visits). Adherence was analyzed as panel data of autocorrelated observations clustered at the patient level, using a random-effects logit model.

A multivariate random-effects logit model was also implemented to assess the influence of socio-demographic and clinical variables on adherence to re-assessment visits among patients participating in the TRRH program. Statistical analysis was performed using Stata 17 [48].

## 3. Results

### 3.1. Socio-Demographic and Clinical Characteristics in the Two Programs

The study included 2198 patients enrolled in the TRRH program between March 2019 and March 2024. The majority of participants were females (75%), aged 60 and above (51%), and without health insurance (75%). HTN was the most prevalent condition, affecting 1542 patients (70%). Additionally, 313 patients (14%) had DM, while 343 (16%) were diagnosed with both conditions. Approximately one-third of patients with HTN and DM were newly diagnosed, with about one-third of HTN patients showing very high BP (≥180/110 mmHg) and nearly half of DM patients presenting very high FBG levels (≥11 mmol/L). A detailed summary of the enrollees’ characteristics is provided in Table 1.

As shown in Table 2, 571 patients were enrolled between July 2023 and March 2024 in the HC program. Almost all the enrollees were females without insurance. Most of the patients (87%) had a diagnosis of HTN (*n* = 499), 33 (6%) had a diagnosis of DM, and 41 (7%) were diagnosed with both diseases. Approximately half of the patients (49%) lived more than 20 km away from the TRRH clinic. Almost one-third of HTN patients had a very high BP level (≥180/110 mmHg), and 36% of DM patients presented the highest level of FBG (≥11 mmol/L).

### 3.2. Follow-Up Adherence and Influencing Factors in the TRRH Program

After 6 months, 18 patients were either dead or discharged or transferred, 767 did not attend the 6-month visit, and 201 of them were traced back later, as they skipped the scheduled visit but attended a subsequent one. Considering the overall period of enrollment from the first visit (42 months), the percentage of patients on follow-up visits (patients attending their scheduled visit over the patients eligible for reassessment) decreased from 50.6% to 38.5% in 6 and 42 months, respectively, accounting for an overall weighted average 42-month adherence rate equal to 40.8% (CI 95%: 39.0–42.6%). The details of the follow-up attendances are explained in Table 3.

Table 4 presents the results of multivariate analyses of factors influencing attendance in the TRRH program. Factors positively associated with higher attendance included having NHIF insurance coverage, being peasant and aged 40–59 years old. In contrast, males and those residing farther from the TRRH clinic were found to have lower attendance rates for scheduled follow-up appointments.

Greater distances were associated with an increasing lower probability of attendance, particularly for patients living more than 80 km from the TRRH clinic.

### 3.3. Follow-Up Adherence in the HC Program

Table 5 summarizes the patient adherence to supervision visits over 10 months in the HC program. Particularly, the overall weighted average 10-month adherence rate increased to 91.6% (CI 95%: 90.4–92.8%), with all patients attending their scheduled visits by months 9 and 10.

## 4. Discussion

This study demonstrated high adherence rates to scheduled follow-up visits after decentralizing the NCD program from hospital-based care to residence-level services. Additionally, data from the first four years of the integrated NCD program, conducted at the hospital level, indicated that the distance from the point of care was the most significant factor influencing adherence to follow-up visits. Our findings reinforced this, showing a clear inverse relationship between distance and adherence, with an OR of 0.17 (95% CI: 0.08–0.36) for distances of 80 km or more.

These results aligned with findings from three similar studies, which considered the feasibility and impact of decentralization from district-level hospitals to primary healthcare centers in SSA countries, namely Malawi, Rwanda, and South Africa. Malawi, Pfaff et al. reported a 70% short-term retention rate of diabetic patients attending follow-up visits after a pilot project decentralized care across eight HCs in four health districts, with no significant differences in adherence and adequate DM and HTN control between hospitals and HCs [41]. Similarly, a South African pilot study, implemented across 10 community NCD clinics, evaluated the short-term impact of decentralization in nurse-led clinics. The short-term adherence rate was approximately 85%, and this improvement was accompanied by enhanced physiological outcomes for patients with NCDs [42]. A study in Rwanda showed that the odds of being retained in the program were lower (OR 0.11, CI 95% 0.02–0.62) among HTN patients accessing three district hospitals in comparison with patients accessing seven peripheral HCs. In addition, for those retained, there was no significant difference in achieving blood pressure targets between those accessing district hospitals and HCs [43].

DM and HTN represent a major public health concern in Tanzania [15,23]. However, healthcare services for such diseases are largely concentrated in urban areas and hospital settings, despite the fact that 4 out of 10 Tanzanians live in rural areas [18].

Adherence to NCD programs must be viewed within the context of the limited awareness of cardiovascular risk among rural populations in SSA countries [17]. Unlike acute conditions such as malaria, managing DM and HTN requires patients to have a basic understanding of the chronic and often asymptomatic nature of these conditions, along with a shift in health-related attitudes and a long-term commitment to therapy adherence and trustworthy relationships with health providers [49].

Notably, health-seeking behaviors through screening, diagnosis, and treatment programs for TB and HIV have been largely assessed in many rural communities in SSA [50,51,52]. However, the approach to NCDs in these populations remains largely underexplored [16,53,54,55], and there is still a need for more efficient and cost-effective interventions, including decentralization to community levels [56]. Lessons learned from TB and HIV programs have demonstrated that distance is a major barrier to the implementation of widespread and effective screening and long-term treatment interventions at the primary care level [57,58,59,60,61]. In Tanzania, high-quality HIV care has been successfully established, with nearly 80% of people living with HIV receiving regular care and 90% achieving viral suppression [62].

In contrast, the long-term, multifaceted, and integrated interventions of DM and HTN remain, nowadays, a significant challenge for the health systems in SSA [16,17,63]. The Pragmatic cluster randomized controlled trial (INTE-AFRICA) and the concurrent process evaluation (INTE-COMM), currently underway in Tanzania and Uganda [56], demonstrated that the integrated management of HIV, HTN, and DM is feasible and impactful. Indeed, some experts proposed that existing HIV and TB platforms could serve as a foundation for implementing and improving primary care services for HTN and DM [64,65,66]. However, the integration of these services is not without challenges. Factors like limited trained healthcare staff, infrastructural gaps, and drugs availability across facilities have affected the success of such integration in some settings, as seen in Ethiopia [64].

From the outset, it is essential to account for economic constraints and ensure the long-term sustainability of such programs [63]. This involves capacity building through task-sharing and task-shifting initiatives aimed at empowering local health personnel and community health workers. Furthermore, strengthening referral systems, establishing diagnostic and treatment protocols that utilize simple, cost-effective technologies, and ensuring efficient transport and logistics for drug supply and distribution are key elements. There is no ideal model, and it is necessary to start with pilot projects, like the WHO PEN-PLUS initiative [36], rigorously assess their outcomes, and refine these interventions to achieve the overarching goal: bridging the final gap between primary healthcare services and the underserved communities that need them most [38,67,68,69]. To enhance the accessibility of prevention, screening, and treatment interventions for HTN and DM in rural settings, a participatory approach to community health promotion and prevention is also crucial [68].

Two recent scoping reviews assessed studies on community-based care models for managing HTN and DM [70,71]. The health system saw benefits such as task sharing among various professionals and expanded access to services, as well as the prevention of other cardiovascular diseases. From the patients’ perspective, the main advantages included greater flexibility in accessing services, along with reduced costs and waiting times. However, significant drawbacks included high dropout rates and the fact that these care models often operated in parallel, rather than being fully integrated.

This achievement is highly demanding, as it needs to develop, support, and enhance the construction of professional training networks, the delivery of innovative healthcare technologies, mainly drugs and diagnostic tools, the strengthening of logistical organization, data management and information sharing, the integration with existing primary health services and platforms. It also requires long-term collaborative programs among a variety of stakeholders who play different roles in local healthcare systems.

The main limitation of the study, which paves the way for future research possibilities, came from its descriptive design, depicting two distinct cohorts of patients and not allowing specific causal inferences. However, this limit was partially offset by the extent of the patients enrolled (2198), data collection period (5 years) and the fact that the patients enrolled in the two programs were largely overlapping.

## 5. Conclusions

This study suggested that bringing primary care and diagnostic services for HTN and DM closer to people living in rural areas may increase program adherence.

Breaking down the barrier of distance and providing primary care and diagnostic services for HTN and DM in rural areas may foster substantial benefits. For patients, it reduces the need to spend time and resources traveling to distant reference points of care where such services are typically concentrated. For the broader community, it fosters regular interactions with healthcare personnel and community health workers, thus strengthening local networks and promoting health awareness and prevention initiatives.

Further research could determine the impact of NCDs integrated models at the lowest levels of the healthcare system by assessing the accessibility to screening, diagnosis and clinical treatment.

## Figures and Tables

**Table 1:** Socio-demographic and clinical characteristics of the enrollees in the TRRH program (*n* = 2198).

		No.	%
Population	Total Population	2198	100%
	Male	548	25%
	Female	1650	75%
Age classes	15–39 years	173	8%
	40–59 years	884	40%
	60–79 years	1065	48%
	80+ years	76	3%
Occupation	Retired/Housewives/Unemployed	305	14%
	Peasant	1438	68%
	Skilled workers	368	17%
Distance from the TRRH	0–19 km	611	29%
	20–39 km	540	26%
	40–59 km	353	17%
	60–79 km	393	19%
	80+ km	175	8%
Health Insurance	no NHIF	1650	75%
	NHIF	548	25%
Diagnosis	Hypertension	1542	70%
	Diabetes Mellitus	313	14%
	Both	343	16%
Disease Awareness	Yes	1505	68%
	No	693	32%
	Already known HTN	1258	67%
	HTN new diagnosis	627	33%
	Already known Diabetes	446	68%
	Diabetes new diagnosis	210	32%
Baseline CVD complications	No complications	1605	73%
	Complications	593	27%
Blood Pressure	BP < 140/90 mmHg	242	13%
	BP ≥ 140/90 mmHg	409	22%
	BP ≥ 160/100 mmHg	603	32%
	BP ≥ 180/110 mmHg	625	33%
Fasting Blood Glucose	FBG < 7 mmol/L	125	20%
	FBG 7–8.9 mmol/L	107	17%
	FBG ≥ 9 mmol/L	93	15%
	FBG ≥ 11 mmol/L	313	49%
Body Mass Index	<18 kg/m^2^	76	4%
	18–24 kg/m^2^	693	34%
	25–29 kg/m^2^	650	32%
	≥30 kg/m^2^	592	29%
Lifestyle	Correct lifestyle	1114	51%
	Uncorrect lifestyle	1084	49%

**Table 2:** Socio-demographic and clinical characteristics of the enrollees in the HC program (*n* = 571).

		No.	%
Gender	Total Population	571	100%
	Male	89	16%
	Female	482	84%
Age	15–39 years	18	3%
	40–59 years	146	25%
	60–79 years	289	51%
	80+ years	118	21%
Distance from the TRRH	0–19 km	0	0%
	20–39 km	294	51%
	40–59 km	134	23%
	60–79 km	96	17%
	80+ km	47	8%
Health Insurance	no NHIF	535	94%
	NHIF	36	6%
Diagnosis	Hypertension	498	87%
	Diabetes Mellitus	33	6%
	Both	40	7%
Disease Awareness	Already known HTN	218	41%
	HTN new diagnosis	163	30%
	Already known Diabetes	157	29%
	Diabetes new diagnosis	41	55%
Blood Pressure	BP < 140/90 mmHg	17	3%
	BP ≥ 140/90 mmHg	157	29%
	BP ≥ 160/100 mmHg	173	32%
	BP ≥ 180/110 mmHg	191	36%
Fasting Blood Glucose	FBG < 7 mmol/L	29	40%
	FBG ≥ 7 mmol/L	10	14%
	FBG ≥ 9 mmol/L	8	11%
	FBG ≥ 11 mmol/L	26	36%

**Table 3:** Patients attending 6-month reassessment visits of the TRRH program (*n* = 2198).

	6 Months	12 Months	18 Months	24 Months	30 Months	36 Months	42 Months
Patients attending their scheduled visit	1008	682	535	426	363	318	265
Patients who were traced back	767	734	701	617	594	497	403
Patients eligible for reassessment §	1976	1644	1424	1182	1051	870	704
% of patients attending their scheduled visit (CI 95%) *	50.6%	40.7%	37.3%	36.7%	35.9%	38.1%	38.5%
	(48.5–52.7%)	(38.4–43.0%)	(34.9–39.8%)	(34.1–39.3%)	(33.2–38.6%)	(35.2–41.0%)	(35.4–41.6%)
Overall weighted average 42-month adherence (CI 95%) *	40.8% (39.0–42.6%)						
Last visit ≤ 210 days before	204	526	737	969	1093	1271	1436
Dead/Transferred/Discharged Patients	18	28	37	47	54	57	58

* Estimates from the random effects logit model (8851 visits among 1976 patients). § Include traced-back patients skipping the scheduled visit but attending a subsequent one.

**Table 4:** Factors influencing attendance reassessment visits. Multivariate random effects logit model (*n* = 8342 visits among 1811 patients) *.

		OR	CI 95%		*p*
Gender	Male	**0.48**	**0.3189**	**0.7345**	**0.001**
	Female	1.00			
Age	15–39 years	1.00			
	**40–59 years**	**2.38**	**1.14**	**4.99**	**0.021**
	60–79 years	1.80	0.83	3.88	0.135
	80+ years	1.46	0.44	4.85	0.540
Occupation	Retired/Unemployed/Housewife	1.00			
	**Peasant**	**2.48**	**1.45**	**4.22**	**0.001**
	Skilled worker	0.76	0.39	1.50	0.432
Health Insurance	no NHIF	1.00			
	**NHIF**	**1.82**	**1.20**	**2.77**	**0.005**
**Distance from TRRH**	**0–19 km**	1.00			
	**20–39 km**	**0.58**	**0.36**	**0.95**	**0.031**
	**40–59 km**	**0.49**	**0.28**	**0.84**	**0.011**
	**60–79 km**	**0.45**	**0.26**	**0.77**	**0.004**
	**80+ km**	**0.17**	**0.08**	**0.36**	**0.000**
Diagnosis	Diabetes Mellitus	1.00			
	Hypertension	0.73	0.43	1.25	0.249
	Both	1.04	0.53	2.03	0.906

* Adjusted for reassessment visit number. In bold character: results with a statistical significance *p* < 0.05.

**Table 5:** Patients attending 1-month supervision visits in the HC program (*n* = 571).

	1 Month	2 Months	3 Months	4 Months	5 Months	6 Months	7 Months	8 Months	9 Months	10 Months
Patients attending their scheduled visit	406	344	293	228	195	172	142	112	77	50
Eligible for follow-up	501	374	309	243	201	179	143	115	77	50
% of patients attending their scheduled visit (CI 95%) *	80.6%	92.0%	94.8%	93.8%	97.0%	96.1%	99.3%	97.4%	100.0%	100.0%
	(77.0–84.1%)	(88.9–84.6%)	(91.8–97.0%)	(90.1–96.5%)	(93.7–98.9%)	(93.7–98.9%)	(96.2–100%)	(92.7–99.5%)	(95.4–100%)	(92.9–100%)
Overall weighted average 10-month adherence (CI 95%)	91.6% (90.4–92.8%)									
Last visit ≤ 60 days	70	197	262	328	370	392	428	456	495	523

* Estimates from the random effects logit model (2605 visits among 501 patients).

## Data Availability

All data used in the manuscript are available upon request by contacting the lead author.
